# Frequency and satisfaction of conventional and complementary or alternative therapies for neuromuscular disorders

**DOI:** 10.1186/s42466-023-00281-5

**Published:** 2023-10-12

**Authors:** Lene Änne Böhne, Corinna Wirner, Benedikt Schoser, Carsten Schröter, Petra Baum

**Affiliations:** 1 Department and Outpatient Department of Neurology, University of Leipzig (UKL), Liebigstraße 20, 04103 Leipzig, Germany; 2 Department of Neurology, Friedrich-Baur-Institute, Ludwig-Maximilians-University, Ziemssenstraße 1, 80336 Munich, Germany; 3 Department of Neurology, Hoher Meißner Clinic, Hardtstraße 36, 37242 Bad Sooden-Allendorf, Germany

**Keywords:** Symptomatic therapies for neuromuscular diseases, CAM, Chronic neuromuscular diseases

## Abstract

**Background:**

Causal therapies are not yet available for most neuromuscular diseases. Additionally, data on the use of complementary or alternative therapies (CAM) in patients groups with a variety of different neuromuscular diseases are rare. This retrospective cross-sectional study aims to record the frequency of use and satisfaction of conventional therapies and complementary or alternative medicine (CAM) in patients with neuromuscular disorders in order to compare them afterwards.

**Methods:**

Patients from the University of Leipzig (Department and Outpatient Department of Neurology), the Friedrich-Baur-Institute (Department of Neurology), the Hoher Meißner Clinic (Department of Neurology) and the German Society for Muscular Diseases (DGM e.V.) were included. The focus of this study has been on patients with chronic neuromuscular diseases. Our data are based on standardised questionnaires in analogue form from three German neuromuscular centres and in digital form from the official website of the German Society for Muscular Diseases. Therapy satisfaction was assessed with the Patient Evaluation of Global Response (PEGR) ranking scale (very satisfactory + 2 to very unsatisfactory − 2).

**Results:**

Of 192 questionnaires analysed, the most common diagnoses were spinal muscular atrophy (n = 42; 21.9%), muscular dystrophies (n = 41; 21.4%) and myotonic dystrophies (n = 38; 19.8%). More than half (n = 112; 58.3%) used both conventional and CAM treatments. Physiotherapy (n = 165) was used most frequently within all treatments with conventional manual (PEGR 1.25, *p =* 0.013; CI 95%) and aquatic therapy (PEGR 1.3, *p =* 0.038) showing significantly higher satisfaction compared to therapy on training equipment. Less-used therapies such as psychotherapy (n = 53; PEGR 1.2) were also satisfactory. Within CAM, massages (n = 96) were the most reported and meditation (PEGR 1.5) was best rated. Massage therapy was significantly more satisfactory than progressive muscle relaxation (*p =* 0.003) and chiropractic treatment (*p =* 0.036). Chiropractic treatment (PEGR − 0.1) was rated most negatively. No significant differences were found between the group of conventional therapies and CAM users regarding social and disease-dependent factors.

**Conclusions:**

Treatment with conventional therapy (manual, aquatic therapy) and some CAM therapies can be recommended if adequately indicated.

## Background

The non-uniform group of neuromuscular disorders affects the musculature, neuromuscular transmission and peripheral nerves [25]. Chronic courses are associated with significant psychological and physical impairments and loss of quality of life. The severity of the impairment depends on the specific disease, its pathogenesis and clinical symptoms, prognosis, and therapeutic options. Although causative therapies are already available for a few neuromuscular disorders (e.g. enzyme replacement for Pompe disease, gene-modifying therapies for spinal muscular atrophies), symptomatic treatments play an essential role, and symptomatic therapies with appropriate conventional medical treatments (e.g. physical therapy) and assistive devices (e.g. walking aids) are paramount.

Conservative therapies include physiotherapy, occupational therapy, speech therapy, psychotherapy and psychological pain management. In this study, physiotherapy was further subdivided into five subgroups: Aquatic therapy, therapy on training equipment, conventional manual therapy and physiotherapy according to the neurophysiological principles of Vojta and Bobath respectively. In particular, aquatic therapy has been described with advantages such as joint protection, no risk of falling and high training adaptability to personal needs in terms of strength and endurance [19].

Serving as a treatment possibility for neuromuscular diseases, occupational therapy, exceptionally “motor-functional and sensorimotor perceptive treatments” (HeilM-RL, version May 17th 2022), focus on maintaining daily routines or basic skills [3].

Speech therapy is prescribed for speech, speech flow, language, voice and swallowing disorders [5]. Especially with regard to neuromuscular diseases, e.g. facioscapulohumeral dystrophy [20] or spinal muscular atrophies [16] and the accompanying dysarthria and dysphagia, logopaedic exercises can be used.

Speech therapy is also important in myotonic dystrophies and muscular dystrophies associated with facies myopathica, as it can help improve social participation and communication without complications by preserving facial expressions [20]. Likewise, logopaedic exercises can be crucial for metabolic diseases such as early-onset Pompe disease, as it supports the maintenance of motor skills for swallowing and speaking [8].

Neuromuscular diseases may also present with psychological symptoms. Thus, fatigue and depression occur more frequently in individuals with muscular dystrophy [2] and myotonic dystrophy type 1 [30]. Therefore, psychotherapy may be beneficial. Possible forms of therapy include psychologically based rehabilitation, psychoeducation in a group setting, or cognitive behavioural therapy [29].

In addition to conventional therapies, complementary and alternative medicine (CAM) is also used for neuromuscular diseases [15]. Complementary medicine comprises therapies used in conjunction with conventional medicine, alternative medicine is used instead of conventional medicine. CAM therapies include alternative medical systems (e.g. Ayurveda, homoeopathy), mind–body interventions (meditation, prayer), biologically based treatments (diets, phytotherapy), body-based treatments (chiropractic measures, massages, acupressure) and energy therapies (Qi Gong, laying on of hands) [15].

CAM usage was investigated in children with Duchenne muscular dystrophy [17] and neuropathy patients [7]. However, to date, studies have yet to analyse and compare both conventional medicine and CAM in parallel regarding several chronic neuromuscular diseases, which will be the aim of this study regarding usage frequency and satisfaction.

## Methods

From three different muscular disease centres (Departments of Neurology at the University of Leipzig; Hoher Meißner Clinic, Bad Sooden-Allendorf; and Friedrich-Baur-Institute, Ludwig-Maximilians University Munich), patients with confirmed neuromuscular diseases as well as members of the German Muscle Society (DGM e.V.) were included. Patients diagnosed with rapidly progressing non-hereditary diseases such as amyotrophic lateral sclerosis, Polymyositis and Guillain-Barré-syndrome were excluded. In addition, "supplements (enzymes/vitamins)" given to patients with Pompe disease were not evaluated as CAM but as conventional enzyme replacement therapy; the data collection took place from December 2019 to July 2021 at mentioned centres for muscular diseases in analogue form and digitally for DGM members using a standardised questionnaire.

The standardised questionnaire consisted of 51 items. Participants were asked by random selection. The survey assessed socio-demographic characteristics, disease symptoms, known diagnosed comorbidities and concomitant symptoms, current active treatment modalities (conventional and CAM therapies) at the time of the survey, patient satisfaction and side effects associated with different therapies.

To compare different therapies' patient satisfaction, we used the Patient Evaluation of Global Response (PEGR), which consists of a ranking scale ranging from very good perceived satisfaction (+ 2) of the treatment to very bad (− 2). We included a list of possible CAM treatments and considered all treatments reported by at least fifteen patients. Regarding conventional medicine, the limit has been set at eight persons.

All patients' frequencies and percentages of conventional and CAM therapy use were calculated via Microsoft Excel 2018 (software package Microsoft 365 for Enterprise, Microsoft Corporation, Redmond, USA). To compare different therapies and calculate other predictors, we used the t-, chi-square-, Fisher-Yates-, Kruskal-Wallis-test and the Bonferroni post-hoc test using SPSS (software package SPSS 1997, IBM, USA).

## Results

A total of 192 questionnaires could be evaluated (98 Department of Neurology, University Leipzig, 37 Hoher Meißner Clinic, Bad Sooden-Allendorf, 49 Friedrich-Baur-Institute, LMU Munich, 8 DGM members).

**Fig. 1:**
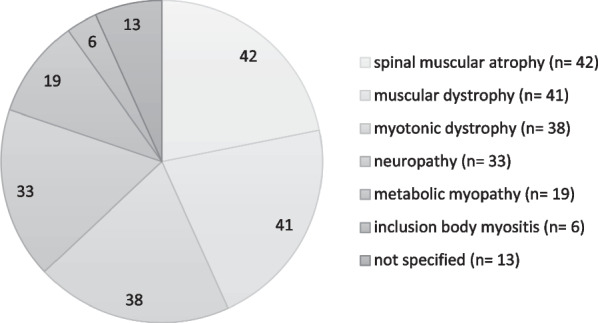
Number of patients regarding different diagnosed disease groups

All diagnoses of neuromuscular diseases are shown in Fig. 1. Demographic data is found in Table 1.

**Table 1:** Sociodemographic and disease related baseline characteristics

Baseline characteristics	
n = 192 (%)	
Age (years): mean (range, standard deviation SD)	50.99 (17–82, 15.38)
Gender	
Male	93 (48.4%)
Female	99 (51.6%)
Ethnicity	
Caucasian	188 (97.9%)
Relationship status	
In a relationship	129 (67.2%)
No current relationship	63 (32.8%)
Professional life	
Employed	83 (43.2%)
Unemployed	24 (12.5%)
Retired	81 (42.2%)
Duration of disease (years)	
Mean (range, SD)	20.46 (1–75, 14.74)
Disability	
Walking not affected	89 (46.4%)
Uses support for walking outside	23 (12.0%)
Wheelchair	16 (8.3%)
Walking distance (metres):	
Mean (range, SD)	
Support-free	1803.77 (1-unlimited, 5388.25)
With support	657.87 (5-unlimited, 887.77)

Regarding neuromuscular diagnoses, spinal muscular atrophy was the most common (n = 42; 21.9%), followed by muscular dystrophies (n = 41; 21.4%) and myotonic dystrophies (n = 38; 19.8%). An average patient would have been 51 years old, probably female (51.6%), while living in a long-term relationship (67.2%) (see Table 1). Many were already retired (42.2%). Overall, the mean duration of the disease was 20 years. The impairment due to the disease was shown by those fractions of patients dependent on a wheelchair (8.3%) and other aids (12.0%). It was also indicated by the proportion of early retirees (n = 54) who retired earlier due to their disease (94.4%).

More than 50% of our survey’s population (n = 112; 58.3%) used conventional therapies in combination with CAM, 61 patients (31.8%) mentioned exclusively conventional medicine. Very rarely, patients focused only on alternative approaches (n = 8; 4.1%) or rather denied any therapy form use (n = 11; 5.7%).

**Fig. 2:**
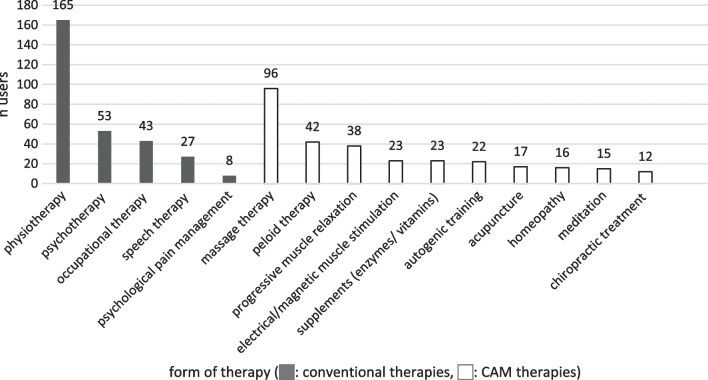
Number of users of conventional therapies and CAM therapies

**Fig. 3:**
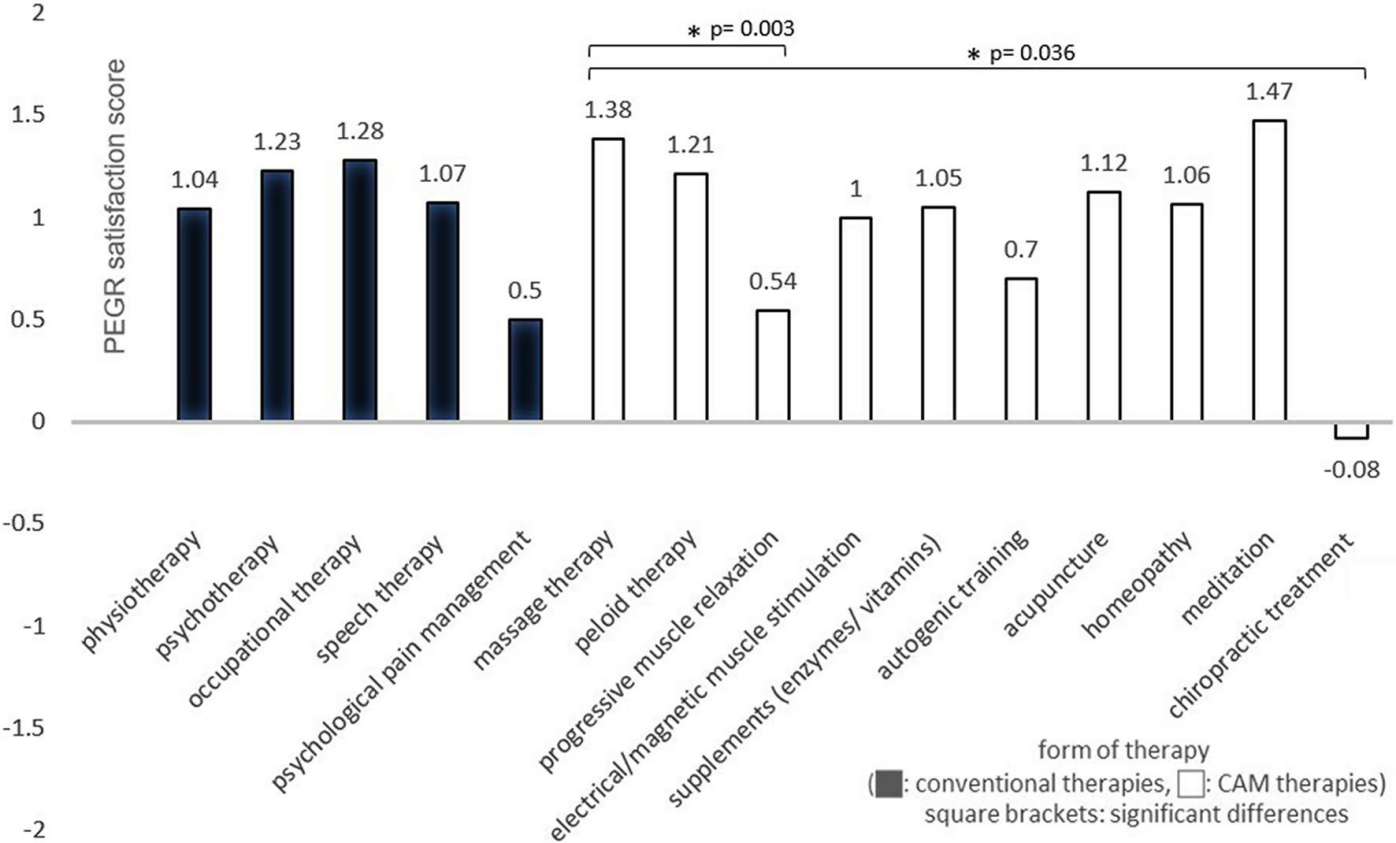
PEGR satisfaction scores of conventional therapies and CAM therapies

**Fig. 4:**
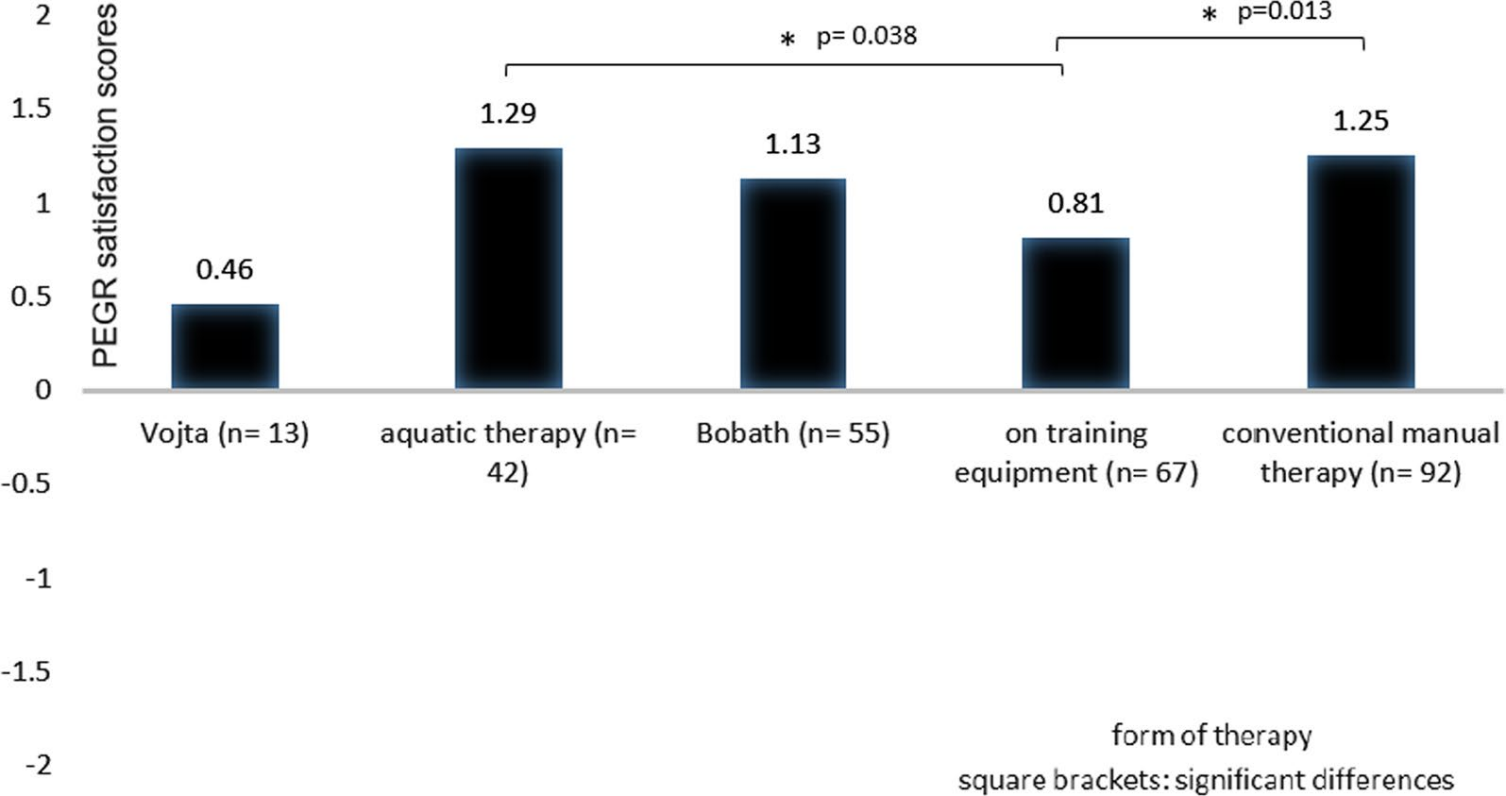
PEGR satisfaction scores of physiotherapy subgroups

**Fig. 5:**
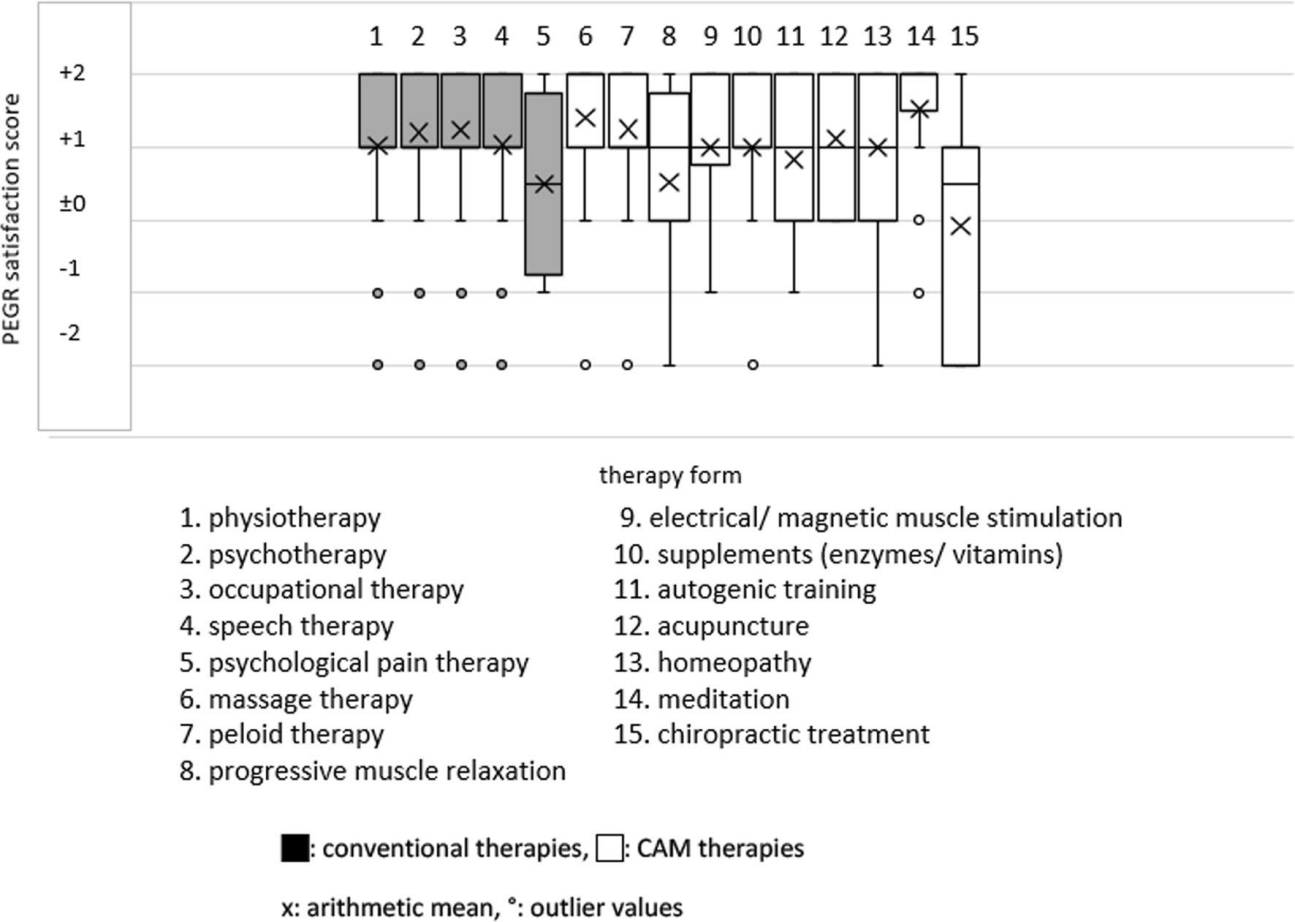
Data distribution of PEGR satisfaction scores of conventional and CAM therapies

**Fig. 6:**
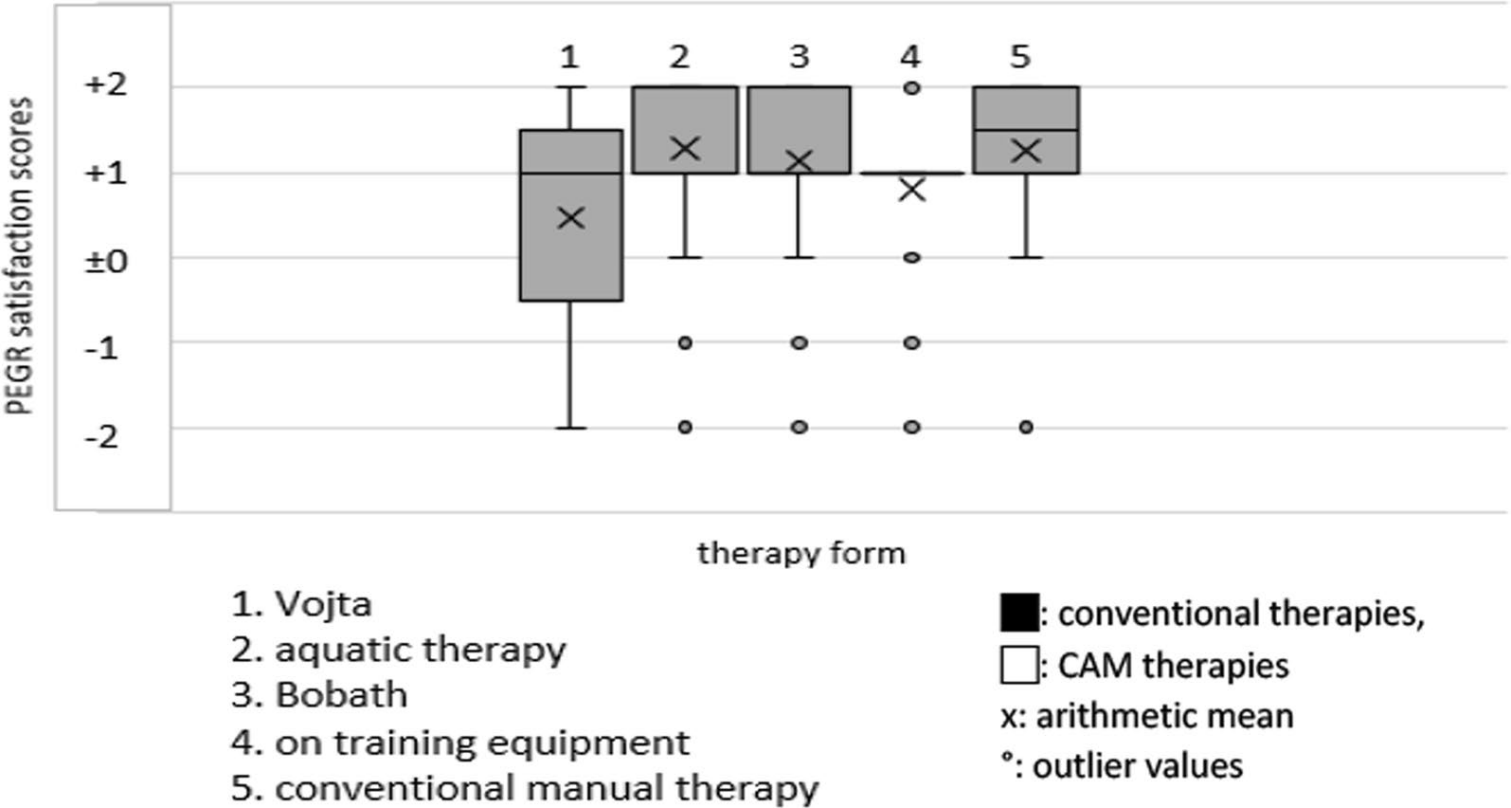
Data distribution of PEGR satisfaction scores of different physiotherapy subgroups

The data collection on conventional therapies (see Figs. 2, 3, 4, 5, 6) revealed that most patients used physiotherapy (n = 165; 85.9%). The most frequent indications showed that the patients used a unit of about 20 to 30 min (n = 115; 59.9%) twice a week (n = 99; 51.6%). The mean value of all stated prescriptions per quarter was 21.9. The most frequently utilised physiotherapy type was conventional manual therapy (n = 92; 47.9%; see Fig. 4); the most satisfactory subgroup was aquatic therapy (PEGR 1.3). In statistical comparison, patients benefitting from aquatic therapy (PEGR 1.3) and conventional manual therapy (PEGR 1.25) proved to be significantly more satisfied than with treatment on training equipment (*p =* 0.038 and *p =* 0.013). The complete data distribution concerning PEGR scores of different physiotherapy subgroups can be found in Fig. 6. Comparing all conventional therapies (physiotherapy, occupational therapy, speech therapy, psychotherapy, psychological pain management) generally yielded no statistically significant results.

Additionally, less frequently used conventional forms such as psychotherapy (n = 53; 27.6%; PEGR 1.2) and occupational therapy (n = 43; 22.4%; PEGR 1.3) proved to be also rated with high satisfaction. Psychological pain management was the least utilised (n = 8; 4.2%; PEGR 0.5) and most unsatisfactory. Most of the patients who provided information went to occupational therapy once a week (n = 26; 13.5%) for a session of 45 min (n = 16; 8.3%), with a mean of 14.4 sessions prescribed per quarter. Speech therapy users mostly reported seeing their speech therapist once a week (n = 21; 10.9) for a session of 45 min (n = 20; 10.4%), with a mean of 13.3 sessions per quarter. No data were provided on the frequency, length and number of units prescribed for psychotherapy and psychological pain therapy.

**Fig. 7:**
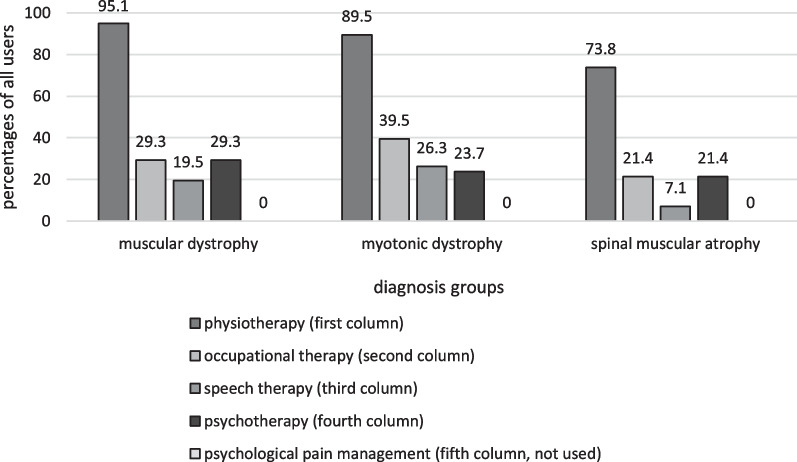
User percentages of three big diagnosed disease groups

Of all neuromuscular diagnosis groups, the three most frequently reported groups (muscular dystrophies, myotonic dystrophies and spinal muscular atrophies) were additionally analysed separately for conventional therapy use. In all three groups, a high percentage used physiotherapy (95.1%; 89.5% and 73.8% retrospectively), although the percentage of patients with spinal muscular atrophies was slightly lower compared to the other groups (see Fig. 7). Less high percentages were present in all groups regarding occupational therapy (29.3%; 39.5% and 21.4%), speech therapy (19.5%; 26.3% and 7.1%) and psychotherapy use (29.3%; 23.7% and 21.4%). The usage of psychological pain therapy was not reported by any of the participants in the three groups. High satisfaction scores were reported for physiotherapy (PEGR 1.2; PEGR 1 and PEGR 1.3) and psychotherapy (PEGR 1; PEGR 1.1 and PEGR 1.2) in all groups. Occupational therapy proved to be less satisfactory (PEGR 0.9; PEGR 0.3 and PEGR 0.6). The data on speech therapy suggested a trend that patients with myotonic dystrophies rated it less satisfactory (PEGR 0.6) than patients with muscular dystrophies (PEGR 1.4) and spinal muscular atrophies (PEGR 1.7).

Within CAM therapies (see Figs. 2, 3), massages stood out in terms of usage frequency (n = 96; 50.0%; PEGR 1.4), while meditation therapy was the most approved treatment (n = 15; 7.8%; PEGR 1.5). Other frequently used treatments were peloid therapy (n = 42; 21.9%; PEGR 1.2), progressive muscle relaxation (n = 38; 19.8%; PEGR 0.5) and enzyme therapies (n = 23; 12.0%; PEGR 1.05). Less frequently utilised but rated with high satisfaction was acupuncture (n = 17; 8.9%; PEGR 1.1). Biofeedback was not used, while chiropractic treatment (n = 12; 6.3%; PEGR − 0.08) frequently received negative feedback. Statistically, it was found that massage therapy was significantly more satisfactory than progressive muscle relaxation (*p =* 0.003) and chiropractic treatment (*p =* 0.036).

A subsequent comparison between conventional and CAM therapies with and among each other (see Figs. 2 and 3) yielded the following results: the most commonly requested therapies were physiotherapy (n = 165, 85.9%) and massages (n = 96, 50%), followed by psychotherapy (n = 53; 27.6%) and occupational therapy (n = 43; 22.3%). Speech therapy, psychological pain management, and all CAM therapies besides massages and peloid therapy were rarely mentioned (< 20%). The most positive responses were found for CAM therapies such as meditation (PEGR 1.5), massage therapy (PEGR 1.4) and peloid therapy (PEGR 1.2), and for conventional subgroups like aquatic therapy (PEGR 1.3) and conventional manual therapy (PEGR 1.25). Chiropractic treatment (PEGR − 0.08) and psychological pain management (PEGR 0.5) received the most negative feedback. There were no statistically significant results regarding the differences between CAM and conventional therapies. The complete data distribution of PEGR satisfaction scores of conventional and CAM therapies can be found in Fig. 5.

The most common reasons for considering complementary or alternative therapies were to benefit from all options (n = 65; 33.9%) and to take an active role in disease management (n = 64; 33.3%).

Financially, it was noticeable that within the group of CAM users (n = 120), a minority reported full reimbursement of CAM therapies (n = 21; 17.5%). In contrast, most patients reported that they received no reimbursement (n = 28; 23.3%) or only partial reimbursement (n = 29; 24.2%) in one year. Own costs for complementary therapies were stated by 13.3% (n = 16) to be more than 100 euros. Of these, six patients (5.0%) indicated additional costs of over 1000 euros. Nevertheless, most patients replying to the question (n = 85; 70.8%) wanted to continue using CAM, even at their own expense (n = 48; 40.0%).

Testing for psychosocial and disease-related predictors of CAM users did not reveal any significant differences (see Table 2).

**Table 2:** Causal variables for using CAM

Variables		Users of CAM n = 120/n/mean (range, SD)	Patients who don’t use CAM n = 72/n/mean (range, SD)	P value CI 95%
Age (years):		50.35 (17–81, 14.62)	52.04 (21–82, 16.62)	0.46
Gender	Female	65	33	0.26
	Male	55	39	
Ethnicity	Caucasian	119	68	0.05
Relationship status	In a relationship	81	47	0.85
	No current relationship	39	24	
Professional life	Employed	59	26	0.15
	Not employed	61	42	
*Disease related characteristics*				
Duration of disease	(years)	21.79 (1–75, 14.42)	18.23 (1–67, 15.10)	0.11
*Impairment (concerning walking outdoors)*				
Uses support		62	29	0.13
Walking not affected		54	40	
Walking distance				
With support		701.08 (5-unlimited, 992.17)	542.67 (20-unlimited, 527.36)	0.45
Without support		1171.67 (1–10,000, 2060.47)	3278.67 (10–50,000, 926.69)	0.23

## Discussion

This study assessed and evaluated conventional and complementary or alternative medical forms (CAM) of therapy for neuromuscular disorders. The data were collected in different neuromuscular centres in Germany. We included 192 patients with confirmed neuromuscular diseases, most frequently spinal muscular atrophy, muscular dystrophies and myotonic dystrophies.

Most studies on this have only examined individual neuromuscular diseases [4, 17] and therapies. There is hardly any data for speech therapy and psychological pain therapy. Former studies that also focused on predictors of CAM therapies concerned patients diagnosed with muscular dystrophies [17, 31] and neuropathies [7].

90% of questioned patients with neuromuscular diseases used conventional treatments. More than 80% were highly satisfied with physiotherapy as their first-choice treatment. Hence, this is in line with the general recommendation to avoid inactivity [10]. However, work on strength and endurance training for neuromuscular diseases such as myotonic dystrophy and Duchenne muscular dystrophy showed no strong evidence of an effect due to mentioned exercise [27]. Still, it is advised to adjust the treatment to the individual disease, considering the intensity of the training and the orientation towards strength or endurance training [10]. Interestingly, aquatic therapy was rated best, and a trial of this physiotherapy type also demonstrated high satisfaction in muscular dystrophies [31]. In addition, conventional manual therapy also stood out regarding usage and patient response. Regarding physiotherapy units, a study on neuromuscular diseases (such as motor neuron diseases and neuropathies) indicated a therapy frequency of two units of approximately 20 min per week [28]. Recommendations or statements regarding a number of units prescribed per quarter were not mentioned. For other conventional therapies, no sources with recommendations on the frequency, length, and number of prescribed units were available.

Occupational therapy was more rarely used in our study (approx. one fifth of the respondents) and, with exceptions, always combined with physiotherapy. Still, this treatment received a high satisfaction rating. Our results are thus in conformity with benefits concerning symptom or deficit reduction in neuropathy patients [23].

Speech therapy was used less frequently (< 20%), yet it was also reported to be highly satisfactory. Logopaedics should be recommended, particularly in swallowing disorders, voice and language [5]. Likewise, a study on speech characteristics in the congenital and childhood-onset forms of myotonic dystrophy type 1 stated that most patients would need speech therapy early on due to symptoms of the mentioned disease [22]. Work on facioscapulohumeral muscular dystrophy and myotonic dystrophy also described that logopaedics could be essential to maintain insufficient nutrition while preventing aspiration [20]. Speech therapy exercises can reduce speech effort and improve intelligibility, thus social participation and quality of life [20].

Psychotherapy was chosen by more than a quarter of all patients and received very positive feedback. Used psychotherapy subtypes were not specified. A higher quality of life during psychotherapy was found in a study on Duchenne muscular dystrophy [24]. Among studies on effective psychotherapy for neuromuscular diseases, cognitive behavioural therapy is frequently mentioned [14, 18, 26]. Studies showed that cognitive behavioural therapy ameliorated fatigue associated with neuromuscular conditions like facioscapulohumeral muscular dystrophy [26] and myotonic dystrophy type 1 [18]. A study on facioscapulohumeral muscular dystrophy found that aerobic exercise and psychotherapy (cognitive behavioural therapy) directed towards the optimisation of everyday activity decreased the replacement of muscle tissue with fat [14]. In addition, work on myotonic dystrophy patients observed improved social involvement and capability to act associated with cognitive behavioural therapy [18]. With regard to other subtypes, a study on a psychological rehabilitation program for muscular dystrophy patients showed that it had no significant effects on activities of daily living, coping and quality of life [1]. A paper on psychoeducation in patients with neuromuscular diseases, among others, was able to demonstrate a significant improvement in mental health and general health perception [6]. However, both sources were interpreted as only weak evidence for the level of effectiveness of their investigated therapies, as they did not have a sufficiently large control group [29].

Contrarily, psychological pain management was named by less than 5% with a meagre PEGR score. There is no literature on neuromuscular diseases and psychological pain therapy.

The comparison of the three most frequent diagnosed disease groups showed generally similar results regarding use and satisfaction. However, it was noticeable that in contrast to individuals with dystrophies affecting the muscles, patients with spinal muscular atrophy used less physiotherapy and less speech therapy. Regarding the speech therapy usage, this may be explained by the treatment of facies myopathica, a symptom more common in muscular and myotonic dystrophies [20].

Complementary therapies were reported by more than 60% of all respondents in our study, for the majority in combination with conventional treatments. In this regard, a survey of muscular dystrophy patients showed that one fifth of all patients received CAM [21]. Also, in a paper on paediatric cases of Duchenne/Becker muscular dystrophy, up to 80% of all guardians sent their children to CAM practitioners [17].

The most mentioned CAM therapies were massages and peloid therapy, which were reported by half of all individuals and more than one fifth of all patients, respectively, with high contentment. Massages were also frequently reported in literature on muscle dystrophy patients [31].

Equally popular among CAM in our survey was the practice of progressive muscle relaxation, however, with a low level of satisfaction. Nevertheless, sources on progressive muscle relaxation as a treatment for neuropathic pain showed that patients experienced a significant reduction in pain and increased contentment [12]. Similarly, another paper on neuropathies showed a substantial improvement in quality of life and a reduction in fatigue associated with progressive muscle relaxation [13]. However, only a small proportion of patients with chronic or hereditary neuropathies are included in our study.

In contrast, another mind-body application, meditation, had the highest satisfaction score and was used more rarely. The treatment of Duchenne and Becker muscular dystrophies with meditation has already been described [17]. Studies on neuropathic pain above also demonstrated a majority of satisfied patients in addition to symptom improvement after using meditation [12, 13].

Enzyme and vitamin therapies were used by a small fraction in our study, though with a very positive response. Usage and beneficial effects on symptom reduction have been described in the literature, notably in neuropathies [4, 7].

Acupuncture was not commonly reported in our study population (< 10%), yet it also showed a high level of satisfaction. Other authors have similarly noted the increased popularity of acupuncture in patients with neuropathies [4, 7].

Chiropractic and homoeopathy received notable negative feedback in our study. In contrast, a review of neuromuscular diseases (including Duchenne muscular dystrophy) in children revealed that chiropractic measures were among the most frequently used treatments and did not have a less satisfactory effect compared to conventional therapies [21].

Referencing social factors including nationality, age, gender and partnerships, CAM and non-CAM users were not significantly different. However, research on Duchenne and Becker muscular dystrophies [31] and neuropathies [7] showed that CAM users were more likely to be white [31] and younger [7]. Disease-related predictors such as disease duration and physical limitation (reliance on assistive devices and highest walking distance) also didn't differ significantly.

In our sample, the duration of illness was in the mean of 20 years, which suggests a highly chronic course of the disease and associated burden. Despite a lack of significance, there was still a trend towards a longer disease duration among users of complementary and alternative medicine, supporting the thesis that especially people with chronic diseases use CAM therapies more often [9, 31]. One fifth of our participants reported using a wheelchair or assistive devices due to their illness. A study on muscular dystrophies also found higher percentages of wheelchair-dependent and mobility-impaired patients among CAM users [31].

The most frequently cited motivations for choosing an alternative treatment method were mentioned by most patients surveyed as wanting to influence disease management and benefit from all available options actively. Likewise, a desire for a holistic therapy that includes conventional and complementary/alternative approaches was described in a paper studying patients with Duchenne muscular dystrophy, among others [21].

Health insurance is only partially reimbursed or not reimbursed by CAM costs in almost 50% of all CAM using patients. This topic wasn’t described in studies on neuromuscular diseases that we included. For patients in our study, a lack of coverage implied raising their finances. Still, many wanted to use complementary and alternative therapies in the future, indicating the perceived quality of CAM.

### Limitations

This work only examines subjective parameters. For a holistic investigation of these therapies, further consideration of objective effects such as muscle degeneration or mobility under therapy would be beneficial.

Also, the assignment to CAM and conventional therapies was sometimes difficult due to overlaps in, for example, massage and manual therapy. That’s why our work is based in particular on the German catalogue of therapeutic products and its exclusion criteria [11].

Finally, the patient group was also inhomogeneous concerning the diagnoses despite similar symptoms.

## Conclusions

More than 80% of all interviewed patients with chronic neuromuscular diseases consulted physiotherapists, benefitting from this conventional treatment. Though aquatic therapy was rated best, conventional manual therapy was most used among physiotherapy subgroups. The first one can be recommended, particularly for patients with chronic neuromuscular diseases, as it allows muscle training while sparing the joints. Based on our data, at least two sessions per week for at least 30 min each should be recommended. Similarly, almost half of all patients mentioned conventional manual therapy as a type of physiotherapy while being rated with a high satisfaction score.

Less frequently named though highly appreciated, were occupational therapy and speech therapy. Our data suggested that the use of speech therapy or occupational therapy should be recommended at least once a week for a session of 45 min. Therefore, a recommendation of prescribing physiotherapy combined with occupational therapy in cases of bulbar symptoms with additional logopaedics was possible.

The positive effects of psychotherapy were confirmed in this study. Thus, psychotherapy should be advised to improve coping with neuromuscular illness [14, 18, 26].

Psychological pain management played a minor role in this cohort, meaning there was insufficient data to recommend this treatment.

CAM was mainly reported in combination with conventional therapies. Massages, peloid therapy and enzyme and vitamin supplements were most frequently used with a high level of contentment. However, progressive muscle relaxation showed a low rate of satisfaction, although being also commonly named.

Meditation and acupuncture were also highly satisfactory for less frequented complementary therapies, with meditation being rated the best. By contrast, chiropractic measures received negative feedback most often. Hence, they cannot be recommended.

Nevertheless, CAM therapies appear to be a valuable complement to conventional medicine when indicated. Comprehensive patient education about complementary therapies is desired by many patients, especially since the costs are usually not covered by health insurance, which is why patients must invest their financial resources.

## Data Availability

The datasets used and analysed during the current study are available from the corresponding author on reasonable request.

## Abbreviations

CAM: Complementary and alternative medicine
CI: Confidence interval
DGM: German society for muscular diseases
LMU: Ludwig-Maximilians-University
PEGR: Patient evaluation of global response (ranking scale)

## References

[1] Ahlström G, Lindvall B, Wenneberg S, Gunnarsson LG (2006). A comprehensive rehabilitation programme tailored to the needs of adults with muscular dystrophy. Clinical Rehabilitation.

[2] Alschuler KN, Jensen MP, Goetz MC, Smith AE, Verrall AM, Molton IR (2012). Effects of pain and fatigue on physical functioning and depression in persons with muscular dystrophy. Disability and Health Journal.

[3] Arts, M., Bernartz, S. & Bouska, C. (2020). *Ergotherapie*. *Ergotherapie im Arbeitsfeld Neurologie: Herausgegeben von Carola Habermann, Friederike Kolster ; mit Beiträgen von Margo Arts [und 49 weiteren]* (C. Habermann & F. Kolster, Hg.). Georg Thieme Verlag.

[4] Baute V, Zelnik D, Curtis J, Sadeghifar F (2019). Complementary and alternative medicine for painful peripheral neuropathy. Current Treatment Options in Neurology.

[5] Bohnert-Kraus, M., & Kehrein, R. (2020).*Dialekt und Logopädie* (1st ed.). Germanistische Linguistik: 248–249/2020. Georg Olms Verlag. 10.1470/9783487422886

[6] Boosman H, Visser-Meily JMA, Meijer J-WG, Elsinga A, Post MWM (2011). Evaluation of change in fatigue, self-efficacy and health-related quality of life, after a group educational intervention programme for persons with neuromuscular diseases or multiple sclerosis: A pilot study. Disability and Rehabilitation.

[7] Brunelli B, Gorson KC (2004). The use of complementary and alternative medicines by patients with peripheral neuropathy. Journal of the Neurological Sciences.

[8] Bulbulia BA, Laher N, Bulbulia R (2018). Pompe disease: Presentation and management of early onset type with perioperative considerations. Journal of Rare Disorders: Diagnosis & Therapy.

[9] Falci L, Shi Z, Greenlee H (2016). Multiple Chronic conditions and use of complementary and alternative medicine among US adults: Results from the 2012 national health interview survey. Preventing Chronic Disease.

[10] Fossmo HL, Holtebekk E, Giltvedt K, Dybesland AR, Sanaker PS, Ørstavik K (2018). Fysisk trening hos voksne med arvelig muskelsykdom [Physical exercise in adults with hereditary neuromuscular disease]. Tidsskrift for den Norske laegeforening: tidsskrift for praktisk medicin, ny raekke.

[11] Guideline on the prescription of remedies in the care of SHI-accredited physicians (Richtlinie über die Verordnung von Heilmitteln in der vertragsärztlichen Versorgung), SGB V §19 para. 7 p. 23 (2011). https://www.g-ba.de/downloads/62-492-2786/HeilM-RL_2021-10-21_iK-2022-04-01.pdf.

[12] Hussain N, Said ASA (2019). Mindfulness-based meditation versus progressive relaxation meditation: Impact on chronic pain in older female patients with diabetic neuropathy. Journal of Evidence-based Integrative Medicine.

[13] Izgu N, Gok Metin Z, Karadas C, Ozdemir L, Metinarikan N, Corapcıoglu D (2020). Progressive muscle relaxation and mindfulness meditation on neuropathic pain, fatigue, and quality of life in patients with type 2 diabetes: A randomized clinical trial. Journal of Nursing Scholarship: An Official Publication of Sigma Theta Tau International Honor Society of Nursing.

[14] Janssen B, Voet N, Geurts A, van Engelen B, Heerschap A (2016). Quantitative MRI reveals decelerated fatty infiltration in muscles of active FSHD patients. Neurology.

[15] Koithan M (2009). Introducing complementary and alternative therapies. The Journal for Nurse Practitioners: JNP.

[16] Kooi-van Es M, Erasmus CE, de Swart BJ, Voet NB, van der Wees PJ, de Groot IJ, van den Engel-Hoek L (2020). Dysphagia and dysarthria in children with neuromuscular diseases, a prevalence study. Journal of Neuromuscular Diseases.

[17] Nabukera SK, Romitti PA, Campbell KA, Meaney FJ, Caspers KM, Mathews KD, Sherlock SMH, Puzhankara S, Cunniff C, Druschel CM, Pandya S, Matthews DJ, Ciafaloni E (2012). Use of complementary and alternative medicine by males with Duchenne or Becker muscular dystrophy. Journal of Child Neurology.

[18] Okkersen K, Jimenez-Moreno C, Wenninger S, Daidj F, Glennon J, Cumming S, Littleford R, Monckton DG, Lochmüller H, Catt M, Faber CG, Hapca A, Donnan PT, Gorman G, Bassez G, Schoser B, Knoop H, Treweek S, van Engelen BGM, Dittrich J (2018). Cognitive behavioural therapy with optional graded exercise therapy in patients with severe fatigue with myotonic dystrophy type 1: A multicentre, single-blind, randomised trial. The Lancet Neurology.

[19] Plecash AR, Leavitt BR (2014). Aquatherapy for neurodegenerative disorders. Journal of Huntington's Disease.

[20] Rösler W, Schwarz E, Tast H, Wellinger I (2012). Logopädische Therapie bei fazioskapulohumeraler Muskeldystrophie und myotoner Dystrophie Typ 1 (Curschmann, Steinert) im Erwachsenenalter [Logopedic therapy of patients with facioscapulohumeral muscular dystrophy and myotonic dystrophy type I.]. Neurologie & Rehabilitation.

[21] Samdup DZ, Smith RG, Il Song S (2006). The use of complementary and alternative medicine in children with chronic medical conditions. American Journal of Physical Medicine & Rehabilitation.

[22] Sjögreen L, Mårtensson Å, Ekström A-B (2018). Speech characteristics in the congenital and childhood-onset forms of myotonic dystrophy type 1. International Journal of Language & Communication Disorders.

[23] Sommer C, Geber C, Young P, Forst R, Birklein F, Schoser B (2018). Polyneuropathies. Deutsches Arzteblatt International.

[24] Travlos V, Downs J, Wilson A, Hince D, Patman S (2019). Mental wellbeing in non-ambulant youth with neuromuscular disorders: What makes the difference?. Neuromuscular Disorders: NMD.

[25] Turakhia P, Barrick B, Berman J (2013). Patients with neuromuscular disorder. The Medical Clinics of North America.

[26] Voet N, Bleijenberg G, Hendriks J, de Groot I, Padberg G, van Engelen B, Geurts A (2014). Both aerobic exercise and cognitive-behavioral therapy reduce chronic fatigue in FSHD: An RCT. Neurology.

[27] Voet NB, van der Kooi EL, van Engelen BG, Geurts AC (2019). Strength training and aerobic exercise training for muscle disease. The Cochrane Database of Systematic Reviews.

[28] Voorn EL, Koopman F, Nollet F, Brehm MA (2019). Aerobic exercise in adult neuromuscular rehabilitation: A survey of healthcare professionals. Journal of Rehabilitation Medicine.

[29] Walklet E, Muse K, Meyrick J, Moss T (2016). Do psychosocial interventions improve quality of life and wellbeing in adults with neuromuscular disorders? A systematic review and narrative synthesis. Journal of Neuromuscular Diseases.

[30] Winblad S, Jensen C, Månsson J-E, Samuelsson L, Lindberg C (2010). Depression in Myotonic Dystrophy type 1: Clinical and neuronal correlates. Behavioral and Brain Functions: BBF.

[31] Zhu Y, Romitti PA, Conway KM, Andrews J, Liu K, Meaney FJ, Street N, Puzhankara S, Druschel CM, Matthews DJ (2014). Complementary and alternative medicine for Duchenne and Becker muscular dystrophies: Characteristics of users and caregivers. Pediatric Neurology.

